# Synthesis and investigation of a hexyl substituted thieno-fused BODIPY derivative as a versatile near-IR fluorophore

**DOI:** 10.55730/1300-0527.3420

**Published:** 2022-03-19

**Authors:** Yusuf ÇAKMAK

**Affiliations:** 1 Research and Development Center for Diagnostic Kits (KITARGEM), Konya Food and Agriculture University, Konya, Turkey; 2 Department of Bioengineering, Konya Food and Agriculture University, Konya, Turkey

**Keywords:** Dye, fluorophore, absorbance, boron dipyrromethene, bioimaging

## Abstract

The synthesis, photophysical and electrochemical properties of hexyl thiophene substituted thieno[*b*]-fused BODIPY structure are reported within this work. One such derivative, HTFBod, has been studied, which is one of the rare compounds that has a maximum absorbance wavelength greater than 750 nm among fused BODIPY compounds. In addition, it preserves its significant spectral properties such as high molar absorptivity and fluorescence quantum yield. Electrochemical characterizations indicated that the compound could be a successful candidate for an organic solar cell donor compound due to its low band gap, which is known to improve the short-circuit current (*J*_sc_) values. The compound could also harvest the near-IR portion of the solar irradiance where maximum solar photon flux exists. In addition, its low LUMO value of -4.13 eV contributes to its air stability. Hexyl substituents provide greater solubility which is required in solution-processable organic photovoltaics and further bathochromic shift when compared to similar compounds. Next, the synthesis was accomplished in five steps with higher yields compared to similar compounds in the literature. Heavy atom-free singlet oxygen production analysis has also been performed while it has been shown that the compound cannot produce singlet oxygen, while this property could be acquired *via* halogen substitution.

## 1. Introduction

BODIPY compounds remain to be popular among various research groups for their versatile properties for the last few decades. These properties may be counted as large molar absorption coefficients, high fluorescence quantum yields, high stability, and derivatization from various sites.[1] Due to their rich chemistry, they can be employed as fluorescent chemosensors [2,3], fluorescent stains for bioimaging purposes [4], photosensitizers for organic photovoltaics [5], laser dyes [6], photodynamic therapy agents [7–9], active compounds in photocatalysis [10] and dyes for theranostic applications [11]. In recent years, there are various studies aiming to increase the number of near IR absorbing and emitting chemical dye compounds for some applications such as photodynamic therapy, noninvasive bioimaging agents, and organic photovoltaics. For example, in organic photovoltaics, it is known that solar irradiance is strong in the near IR region, therefore harvesting these is highly important to obtain highly efficient devices [12]. Primary BODIPY structures have been absorbing and emitting in the visible range around 500 nm. To shift these to higher wavelengths various strategies have been employed up to date. Extending the *π*-electron conjugation through the *α*- and β- positions [13,14], aza-BODIPYs [15], and fused BODIPY[16] structures are among the most popular strategies. Fused BODIPY structures are classified as [*a*]-fused, [*b*]-fused and zig-zag-fused structures [17]. It has been observed that [*b*]-fused structures have more stabilization on HOMO and LUMO levels compared to the [*a*]-fused structures [17].

In the recent decade, there have been various reports [5,16,18–27] on [*b*]-fused BODIPY dyes for different applications which were recently reviewed [17]. Among these, heterocycle fused compounds attracted much attention, including thiophene and furan fused structures. Although these studies have enriched the properties of primary BODIPY dyes, more studies are required to improve the synthesis, photophysical properties and understand the structure-function relationship for their use in wider application areas. Due to these reasons, a novel hexyl substituted fluorophore based on [*b*]-thiophene fused BODIPY structure was developed and its photophysical, electrochemical properties, and singlet oxygen producing ability were investigated. Recently, our group have also investigated another fused BODIPY compound, namely, [*a*]-benzofused derivative [11]. In that research, we have successfully applied it on HeLa cells for phototoxic effect by comparing it with a novel distyryl derivative, besides exploiting other properties.

## 2. Materials and methods

### 2.1. General methods

All chemicals and solvents purchased from Aldrich, Acros, or TCI were used without further purification unless otherwise stated. ^1^H-NMR and ^13^C-NMR spectra were recorded using a Bruker DPX-400 in CDCl_3_ with TMS as an internal reference. Column chromatography of all products was performed using Merck Silica Gel 60 or Silicycle (particle size: 0.040–0.063 mm, 230–400 mesh ASTM). Reactions were monitored by thin layer chromatography using fluorescent coated aluminum sheets.

Absorption spectrometry in solution was performed using Agilent Cary 60 spectrophotometer. Steady state fluorescence measurements were conducted using an Agilent Eclipse spectrofluorometer. Solvents used for spectroscopy experiments were spectrophotometric grade. Fluorescence quantum yields were calculated by using the method in the literature [28] using the reference dye Zinc phthalocyanine excited at 650 nm in pyridine as the reference fluorophore (Φ_f_ = 0.3) [29].

Mass spectrometry (HRMS) measurements were performed using Agilent LC/MS-High Resolution Quadrupole Mass Time-of-Flight (Q-TOF) or Time-of-Flight (TOF).

Cyclic voltammetry was recorded on a Gamry Interface 1010E instrument in a single-component cell under a nitrogen atmosphere. A typical three-electrode configuration with a glassy carbon as working electrode, platinum wire as counter electrode and a saturated calomel electrode (SCE) as a reference electrode. Potentials were measured versus SCE and referenced to ferrocene as an internal standard (E°(Fc/Fc^+^ ) = −4.78 eV to vacuum). The measurements were performed with a scan rate of 100 mV.s^−1^ in degassed dichloromethane and the addition of tetra-n-butylammonium hexafluorophosphate (TBAPF, 0.1 mol.L^−1^) as electrolyte. The compounds were used as 1 mM concentration.

Singlet oxygen measurements were measured according to the recently reported literature [11].

### 2.2. Synthesis of molecules

Molecules 2 and 3 were synthesized according to the literature [30] with the yields shown on Scheme.

#### Compound 4

Methanol (50 mL) was degassed with N_2_ gas for 15 min. Bithiophene aldehyde 3 (2 g, 6.85 mmol, 1 eq) was dissolved in degassed methanol (17 mL) and ethylazidoacetate (2.65 g, 20.55 mmol, 3 eq) was added. Separately, sodium methoxide (1.1 g, 20.55 mmol, 3 eq) was dissolved in degassed dry methanol (17 mL). The first flask was cooled to 0 °C and a solution of sodium methoxide was added dropwise in half an hour and further stirred for another half an hour at this temperature. Then, at room temperature stirring was continued for 4–6 h and the reaction was stopped upon tracking the formation of the product with TLC (Hexane:Ethyl Acetate, 6:1). Then, sat. NH_4_Cl was added to the reaction and a precipitate was formed. After the filtrate was removed, the precipitate was washed with ethyl acetate (50 mL) and extracted with water (50 mL). Then organic phase was dried with Na_2_SO_4_ and the solvent was evaporated. Then, the compound was dissolved with toluene (50 mL) and degassed with N_2_ for 15 min, and then refluxed at 120 °C for 1.5 h. The solvent was then evaporated and the pure compound was obtained after flash column chromatography purification (Hexane: ethyl acetate, 6:1). Dark yellow color in 28% yield. ^1^H NMR 400 MHz, CDCl_3_, ppm: (d) 9.02 (br s, 1H), 7.07 (m, 1H), 7.03 (m, 1H), 6.96 (m, 1H), 6.70 (m, 1H), 3.90 (s, 3H), 2.81 (m, 2H), 1.67 (m, 2H), 1.1–1.5 (m, 6H), 0.90 (m, 3H). ^13^C NMR (101 MHz, CDCl_3_) δ 175.86, 146.43, 141.97, 135.59, 134.42, 130.00, 125.12, 124.05, 110.09, 108.13, 106.70, 51.96, 31.79, 30.44, 28.98, 22.80, 14.77, 14.32. (HRMS)^−^; calculated for C_18_H_20_NO_2_S_2_^−^ (M–H)^−^ as 346.0941, found 346.0956.

#### Compound 5

Compound 4 (241 mg, 0.693 mmol, 1 eq) and NaOH (580 mg, 14.55 mmol, 21 eq) were dissolved in ethanol (12 mL) and water (6 mL). The reaction mixture was refluxed for 1 h and then cooled to room temperature. By the addition of 6 M HCl, the pH was adjusted to 3 and then the formed precipitate was filtrated. The precipitate was dissolved with ethyl acetate and after drying with Na_2_SO_4_, the solvent was evaporated. No further purification was performed. Off-white solid with 78% yield. ^1^H NMR 400 MHz, DMSO-d_6_, ppm: (d) 11.6 (br s, 1H), 7.07 (m, 1H), 6.97 (s, 1H), 6.75 (m, 2H), 2.75 (m, 2H), 1.60 (m, 2H), 1.4–1.1 (m, 6H), 0.85 (m, 3H). ^13^C NMR (101 MHz, DMSO) δ 163.05, 145.40, 141.81, 139.06, 135.88, 126.00, 123.94, 122.30, 108.04, 106.90, 31.73, 31.65, 30.10, 28.79, 22.73, 14.60. (HRMS)^−^; for (M–H) ^−^ C_17_H_18_NO_2_S_2_^−^ calculated as 332.0784, found as 332.0798.

#### HTFBod

Compound 5 (50 mg, 0.15 mmol) was dissolved with trifluoroacetic acid and stirred at 40 °C for half an hour. Subsequently, trifluoroacetic anhydride (40 *μ*L) was added and heated to 80 °C and stirred for 45 min at this temperature. Then, after cooling to room temperature, sat. NaHCO_3_ in ice-water was slowly added attentively (10 mL). Then, the formed dark green precipitates were filtrated, and the precipitates were dissolved with DCM and dried with Na_2_SO_4_. And the solvent was evaporated. Then, the compound was dissolved in toluene (5 mL) and triethylamine (0.2 mL) and boron trifluoride diethyl ether (0.2 mL) were added. The reaction flask was stirred at room temperature for 1 h and the solvent was evaporated and purified by column chromatography (DCM: Hexane, 1:1). Dark green color with 23% yield. ^1^H NMR 400 MHz, CDCl_3_, ppm: δ 7.28 (d, J = 3.7 Hz, 2H), 7.20 (s, 2H), 7.16 (s, 2H), 6.79 (d, J = 3.8 Hz, 2H), 2.85 (t, J = 7.6 Hz, 5H), 1.71 (p, J = 7.4 Hz, 5H), 1.46–1.30 (m, 15H), 0.95–0.84 (m, 7H). ^13^C NMR (101 MHz, CDCl_3_) δ 160.58, 155.31, 151.94, 138.84, 135.41, 134.21, 128.07, 126.36, 124.48, 117.80, 108.18, 31.74, 31.61, 30.77, 29.93, 28.96, 22.78, 14.29. (HRMS)^−^; for (M–H)^−^ C_34_H_33_BF_5_N_2_S_4_^−^ calculated as 703.1545, found as 703.15386.

## 3. Results and discussion

### 3.1. Synthetic studies

For the synthesis of HTFBod, five steps of synthesis were employed. In the beginning, it was started with commercially available 2,2′-bithiophene. By using hexanoyl chloride and tin (IV) chloride in benzene, a hexanoyl unit was attached. Later, it was reduced to the alkyl form by using LiAlH_4_ and AlCl_3_ in diethyl ether to form compound 2 in 75% yield. Next, it was formylated by using the Vilsmeier-Haack reaction conditions in 67%. These two steps were accomplished according to the literature [30]. The key chemical ethylazidoacetate was synthesized in our laboratory by using ethyl bromoacetate and sodium azide *via* substitution reaction by refluxing the reaction mixture for 4 h in acetone. Then, compound 3 was reacted with ethylazidoacetate in basic conditions in dry methanol through Hemetsberger-Knittel indolization reaction in two steps. The first intermediate is prone to decomposition, therefore, cyclization reaction in toluene was accomplished to obtain a more stable compound 4 in 28% yield. Similar thiophene-substituted fused-thiophene BODIPY derivative has also been synthesized by You group [19,20]. However, they followed a different route: they first synthesized a bromo thieno-pyrrole-fused BODIPY and attached the edge thiophene groups in the later stage through Suzuki coupling and they reported the above-stated indolization reaction yield as 5% in their most recent study to form the bromo substituted thieno-pyrrole. Then, with this strategy, it was proposed a better yield of synthesis of this key intermediate by starting with alkyl-bithiophene rather than attaching the thiophene subsequently through Suzuki coupling.

After obtaining compound 4, it was employed basic hydrolysis to obtain compound 5 in decent yield and without purification. In the next step, compound 5 was reacted with trifluoroacetic acid and trifluoroacetic anhydride. The former chemical is used for decarboxylation reaction, while the latter has been used to form meso-trifluoromethyl substitution. After the formation of the dipyrromethene derivative boron trifluoride diethyl ether complex and triethylamine were added in dichloromethane to form the HTFBod in 23% yield. ^1^H, ^13^C NMR and high-resolution mass spectroscopy characterizations were employed to confirm the structures.

### 3.2. Photophysical characterizations

**Figure 1:**
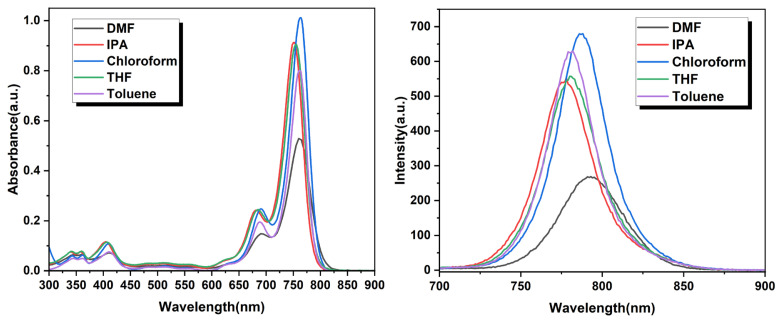
Absorbance (left) and fluorescence (right) spectra of HTFBod in different solvents (5 *μ*M).

To characterize this near IR dye, several studies were employed. First, absorbance spectroscopy studies were investigated in various organic solutions with 5 *μ*M concentration (Figure 1). Thanks to the bis-hexyl units, the compound is well-soluble in organic solvents. The maximum absorption wavelength of HTFBod in chloroform is 764 nm. This compound can absorb between 650 and 810 nm range. The molar absorptivity of this compound has been found as 202,000 M^−1^cm^−1^ in chloroform (Table 1). The maximum fluorescence in the same solvent was obtained at 788 nm. Both the absorbance and emission spectra of this compound are quite sharp with small Stokes shift. A similar observation was also observed by the Suzuki group with Keio Fluor dyes for the [*b*]-furan-fused BODIPY compounds [25]. The full width at half maximum (fwhm) values is similar between these two studies. Then, the fluorescence quantum yield was calculated as 0.11 by using reference dye zinc phthalocyanine. Therefore, since the HTFBod has moderate fluorescence, it could be utilized for bioimaging/imaging purposes. Absorbance and fluorescence characteristics of this dye were also accomplished in four other solvents: dimethylformamide, iso-propyl alcohol, toluene, and tetrahydrofuran (Table 1). It was observed that the compound has similar characteristics in terms of absorbing-emission wavelength range in nonpolar and polar solvents: the absorbance maxima are in 750–760 nm range and emission in 780–790 nm range. The fluorescence has not been disappeared in polar media.

There are few reports on compounds which have maximum absorbance in the region where HTFBod absorbs among fused BODIPY structures. Kubota, Matsui synthesized [*b*]-thiophene fused BODIPY compounds with dimethylaniline attached to the thiophene unit to obtain highly redshifted compounds with 783 and 813 nm absorption maxima due to the strong electron donating effect of the amino unit [18]. In comparison with another related study by the You group, where they investigated thiophene substituted thiophene-fused BODIPY structure [20], it was observed 30 nm bathochromic shift with HTFBod due to the electron donating effect of the alkyl (hexyl) units. The photophysical properties of HTFBod set it in one of the rare fluorophores, that have absorption maxima greater than 750 nm among the fused-BODIPY structures. As far as our knowledge, there is no such compound which has maximum absorption (*λ*_max_) larger than 750 nm and as high molar absorptivity and fluorescence quantum yield than HTFBod [17,31].

Previous studies have shown that attaching electron withdrawing units to the meso position and electron donating units to the 2- and 3- positions of the BODIPY core is a quite useful method for shifting the absorbance wavelength to the red end. Therefore, the employment of electron deficient trifluoromethyl unit and electron-rich bithiophene unit attachment seems to be an effective way of obtaining near IR dyes. In comparison to the similar compound by D’Souza and You, it has been observed that substitution with thiophene rather than phenylene unit has *ca*. 60 nm bathochromic (709 nm vs. 764 nm) [19].

### 3.3. Electrochemical characterization

**Figure 2:**
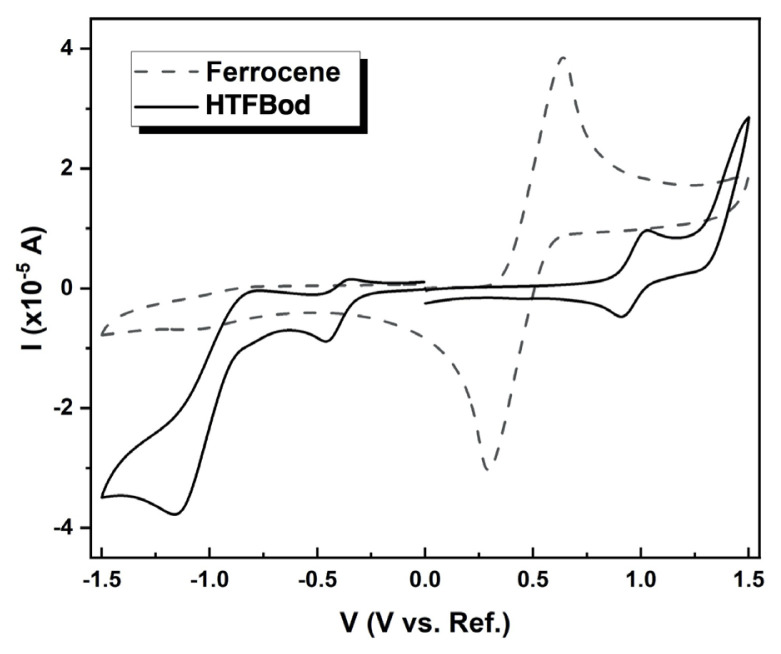
Scans of HTFBod and Ferrocene measured in CH_2_Cl_2_/Bu_4_PF_6_ (0.1 M) versus SCE (scan rate 100 mVs^−1^).

Synthesized near-IR dye HTFBod was examined by cyclic voltammetry (CV) to obtain the oxidation and reduction potentials and the corresponding frontier orbital energy levels (HOMO and LUMO energy levels) and the bandgap. The measurements were accomplished in dichloromethane by using a saturated calomel electrode (SCE) as a reference electrode (The ferrocene/ferrocenium (Fc/Fc^+^) couple was also measured separately as a redox standard) [32]. The voltammograms of HTFBod and ferrocene have been shown in Figure 2.

For the HTFBod, scans were performed between +1.5 and −1.5 V, and one reversible oxidation peak was observed in this region at about +0.89 V, showing a stable Bodipy cation based on the earlier studies [30,33]. Two irreversible reduction waves are also observable with onsets of −0.32 V and −0.84 V.

By using the half-wave oxidation and reduction potentials of the BODIPY compound, the HOMO and LUMO energies have been calculated with the potential of Fc/Fc^+^ as a reference energy of E_(HOMO)_ = −4.78 eV [32,34]. For the HTFBod, HOMO is estimated at −5.34 eV, while the LUMO is −4.13 eV (Table 2). The corresponding bandgap from the cyclic voltammetry measurement was calculated as 1.21 eV. The optical bandgap was also estimated from the onset of absorbance spectrum by the equation given in the literature as E_g_ = 1242/*λ*_onset_ and calculated accordingly as 1.53 eV [35]. The bandgap estimated from the absorbance spectrum is a bit larger than the value obtained from the CV spectrum. Similar observations have also been recorded in other studies [30,32]. Thin-film absorbance data may give closer values.

Decreasing the LUMO energy level of dyes/fluorophores is an efficient way of designing air-stable compounds and is desired for many applications such as organic photovoltaics (OPV) [36]. The presence of a very low LUMO energy level of HTFBod of −4.13 eV is significant in this aspect. Furthermore, the energy levels of the frontier orbitals indicate that HTFBod can be a suitable candidate for organic solar cell donor material for low band gap compounds. It is known that low band gap compounds produce increased short-circuit current (*J*_sc_) which increases the efficiency [37]. However, frequently used organic solar cell acceptor material PC_61_BM and PC_71_BM have LUMO energy levels around −4.0 eV (The LUMO of the PC_61_BM was measured as −4.09 eV) [30] and this value should be lower than the donor compound’s LUMO energy level, which is −4.13 eV. Therefore, compatible with this data other nonfullerene acceptor compounds would suit. Otherwise, some modifications may also be accomplished on HTFBod, such as insertion of phenyl groups instead of trifluoromethyl unit in the meso position. Our observations on the BODIPY core suggest that electron withdrawing groups on this position stabilize the LUMO energy level.

As a successful application of fused BODIPY structures to OPV, Ma and Leo group have synthesized [*b*]-furan fused BODIPY structures and fabricated organic solar cell devices using these compounds as donor compounds by vacuum deposition method and achieved one of the highest efficiencies within BODIPY-based compounds in tandem devices (10%) [5].

### 3.4. Singlet oxygen formation studies

Singlet oxygen generation without using heavy halogen atoms has been extensively studied over the last 10 years [4,7,38,39]. Fused BODIPY structures have also been studied in photodynamic therapy methods for both halogenated [21] and halogen-free versions. Interestingly, in the halogen-free versions, You [19] and Nguyen, Yoon [23] groups have published several thiophene fused structures and have observed singlet oxygen formation in some of the derivatives. They have noted that both the meso substituent and the unit substituted directly to the thiophene play a role. It was observed that for ^1^O_2_ production ability, when the trifluoromethyl group attached to the meso position, the substituent attached to the thiophene unit should not be electron rich.

**Figure 3:**
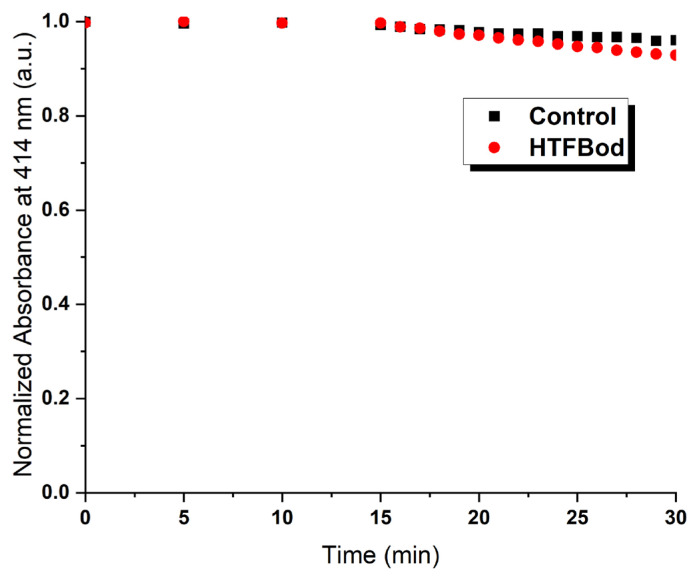
Time-dependent decrease in normalized absorbance (414 nm) of DPBF as a result of oxidation by HTFBod (5 *μ*M) in aerated IPA upon irradiation with 700 nm LED array.

In this case, it was studied the singlet oxygen formation studies with HTFBod to test if it produces singlet oxygen or not. To perform experiment by chemical methods diphenylisobenzofuran (DPBF) as a singlet oxygen scavenger compound was used in organic solvent isopropanol. By using HTFBod and the LED irradiation (700 nm) in an air bubbled solution, the change in absorbance spectrum of the scavenger compound was measured (see Figure 3). However, has not been observed any significant decrease in the absorbance, thereby it was concluded that the compound is not an efficient ^1^O_2_ producer.

The result is also coherent by the observations of the previous study [19]. Attachment of electron-rich hexyl-thiophene structure to the fused thiophene BODIPY unit is expected not to produce ^1^O_2_. They have also estimated that the energy level difference between the HOMO and the LUMO should be at least larger than 1.53 eV. However, in our case, this value was estimated as 1.21 eV. Therefore, producing ^1^O_2_ with nonhalogenated near-IR absorbing compounds becomes more ineffective as the absorption wavelength increases. However, it is possible to produce by halogen substitution [22].

## 4. Conclusion

In this study, the synthesis and investigation of a novel derivative of [*b*]-thiophene fused BODIPY structure were employed. Fused BODIPY structures could be very versatile building blocks for various application areas. This type of structure successfully employed in organic photovoltaics (both dye-sensitized and organic solar cells), photodynamic therapy, and bioimaging studies. Upon investigating various methods to obtain fused BODIPY structures using Hemetsberger-Knittel indolization reaction, it was performed five steps of synthesis starting from the commercially available and cheap 2,2′-bithiophene structure. It has been shown that by using inexpensive chemicals it is possible to reach thiophene substituted thiophene-fused BODIPY structures in a more efficient way compared to the similar compounds in the literature. With an absorbance maximum at 764 nm and emission maxima at 788 nm (in CHCl_3_) it is one of the most redshifted fused compounds. In addition, it has an acceptable fluorescence quantum yield of 0.11, which is almost zero in most of the other near-IR fused compounds (*J*_max_ > 750 nm) [19,20]. Therefore, HTFBod is one of the rare fused compounds that could be used in this wavelength range. Investigation of the electrochemical studies revealed that the low band gap of the HTFBod can be suitable for organic photovoltaics applications since low band gap compounds have higher *J*_sc_ values. Furthermore, near IR absorbing compounds are desired in OPV studies because of the fact that the maximum solar photon flux is located between 600 and 800 nm [40]. However, more suitable nonfullerene acceptors should be employed due to the low LUMO energy level of the compound. Alternatively, in order to increase the LUMO level of the HTFBod, it is proposed to use an aromatic unit instead of trifluoromethyl unit in the meso position of the BODIPY. Our studies based on developing fused structures for applications in various areas are currently under study in our research group.

## SUPPLEMENTARY MATERIAL













ESI-HRMS spectrum of compound **4**

ESI HRMS spectrum of compound **5**

ESI HRMS spectrum of compound **HTFBod**

## Figures and Tables

**Scheme:**
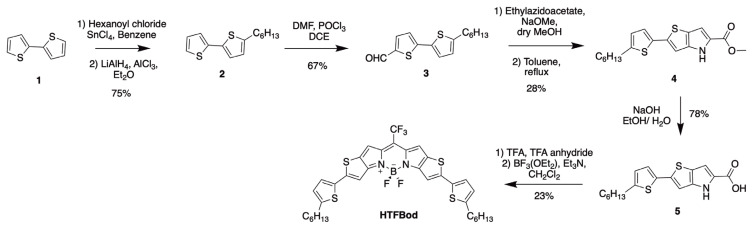
Synthesis of target molecule HTFBod.

**Table 1:** Spectroscopic data for HTFBod in various solvents.

Compound	λ_max_ (abs) nm	λ_max_ (ems) nm	e_max_ (M^−1^cm^−1^)	Dn (cm^−1^)	Solvent	Φ_f_^a^
HTFBod	764	788	202,000	399	CHCl_3_	0.11
	752	778	182,000	444	IPA	
	756	780	180,000	407	THF	
	762	779	160,000	286	Toluene	
	760	793	106,000	548	DMF	

a Fluorescence quantum yield was measured in chloroform and calculated by using Zinc phthalocyanine excited at 650 nm in pyridine as the reference fluorophore (Φ_f_ = 0.3)[29]

**Table 2:** Electrochemical data of the HTFBod, and calculated HOMO-LUMO energy values.

Molecule	E_ox_^onset^(V)^a^	HOMO (eV)^b^	E_red_^onset^(V)^a^	LUMO (eV)^b^	E_g_(eV)^c^	E_g_ (eV)^d^
**HTFBod**	+0.89	−5.34	−0.32, −0.84	−4.13	1.21	1.53

Half-wave potentials of the first oxidation and first reduction, measured in CH_2_Cl_2_/Bu_4_PF_6_ (0.1 M) versus SCE, scan rate 100 mVs^−1^, with Fc/Fc^+^ as standard was measured separately and E_ox_^1/2^ (Fc) was found as +0.33 V. [b] E_HOMO_(Fc) = −4.78 eV, E_HOMO_ = −4.78+ (E_ox_^1/2^(Fc)−(E_ox_^1/2^)), E_LUMO_ = −4.78+(E_ox_^1/2^(Fc)−(E_red_^1/2^)). [c] HOMO-LUMO difference as obtained from CV. [d] based on spectroscopic data in solution.
