# Supplementary figures and images for: Synthesis and investigation of a hexyl substituted thieno-fused BODIPY derivative as a versatile near-IR fluorophore
Source: Turk J Chem. 2022 Mar 19;46(4):1120–7. doi: 10.55730/1300-0527.3420 (PMC10395744; doi:10.55730/1300-0527.3420)

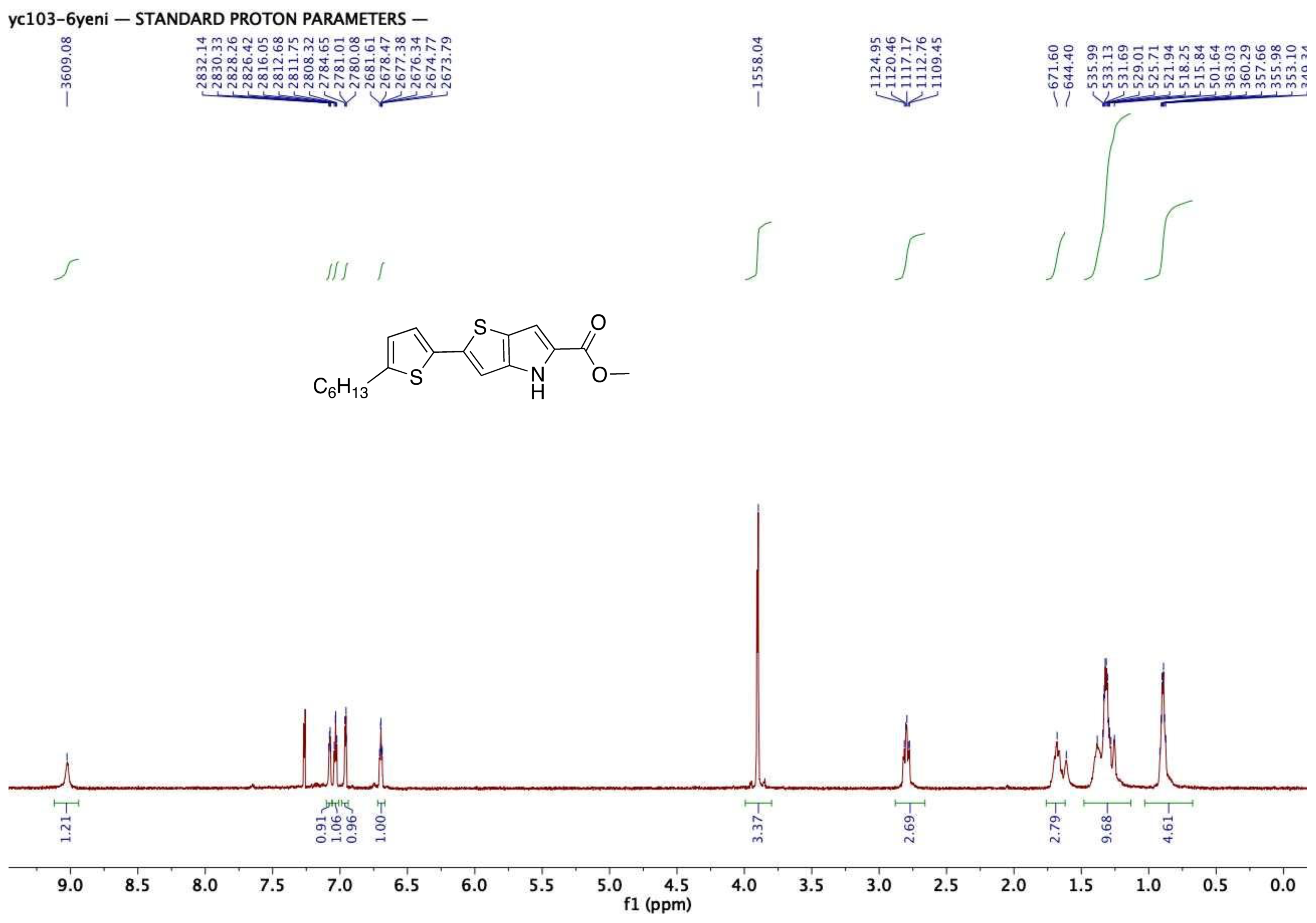

Supplement: Supplementary file 1 [file turkjchem-46-4-1120s1.tif]

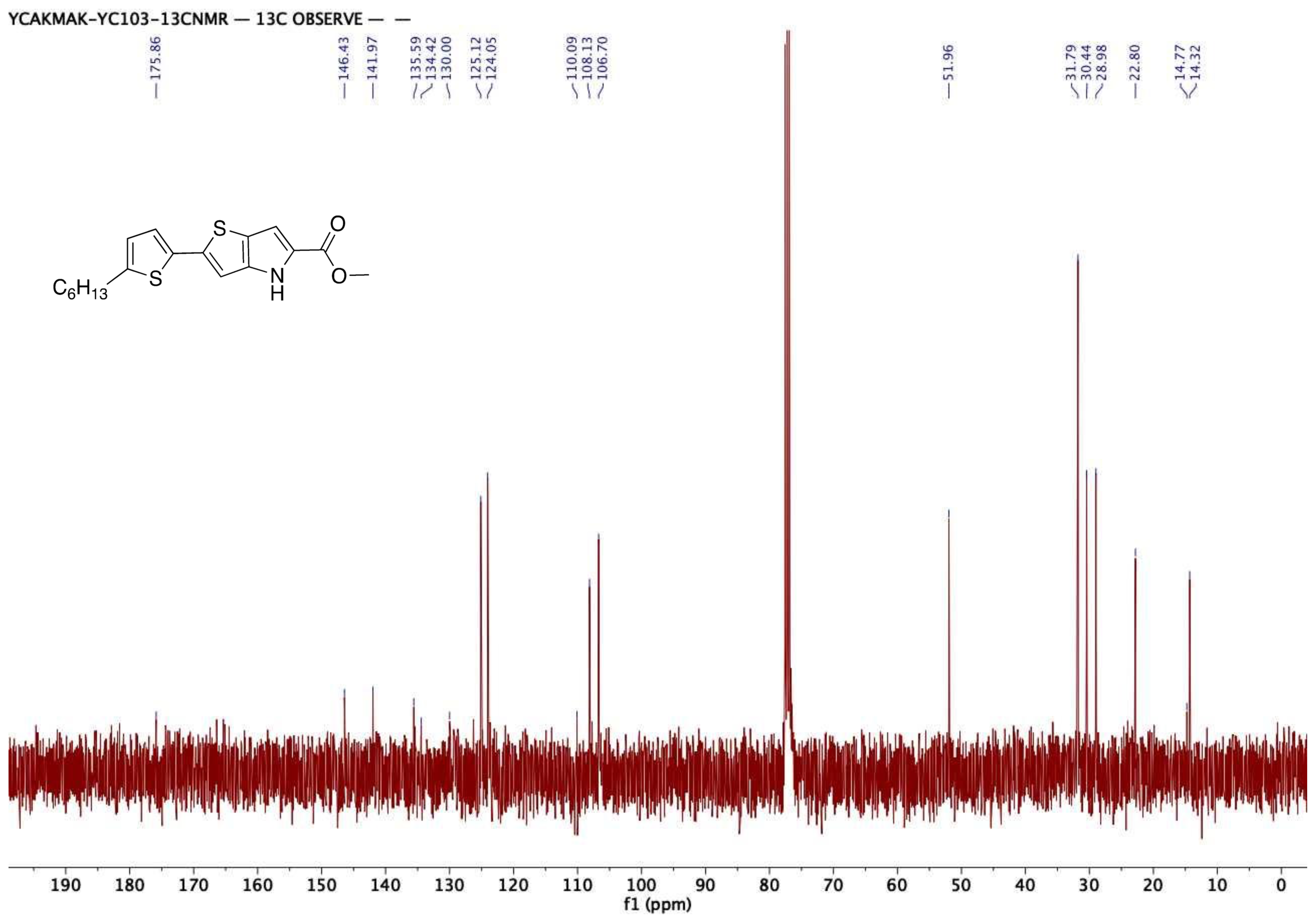

Supplement: Supplementary file 2 [file turkjchem-46-4-1120s2.tif]

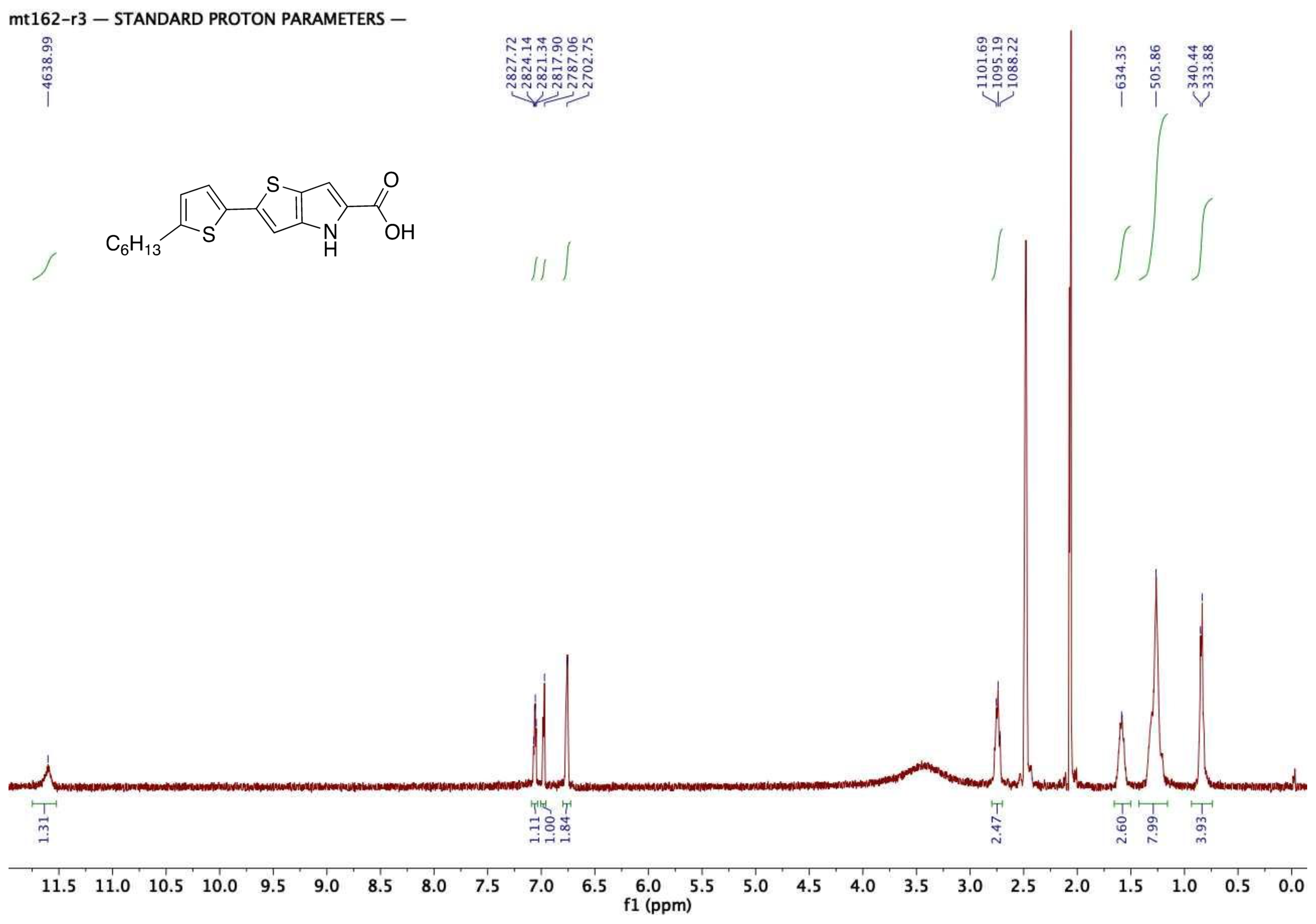

Supplement: Supplementary file 3 [file turkjchem-46-4-1120s3.tif]

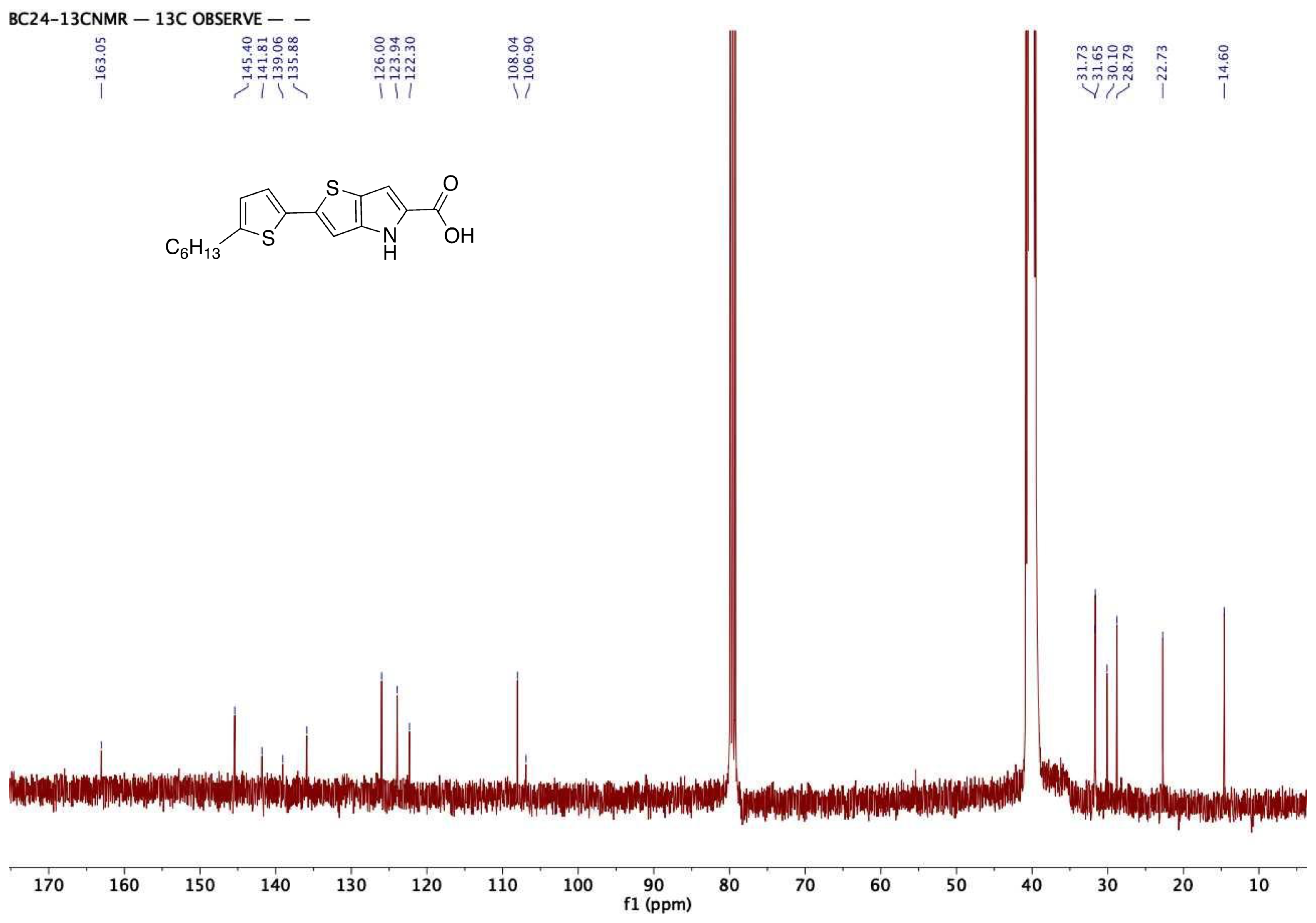

Supplement: Supplementary file 4 [file turkjchem-46-4-1120s4.tif]

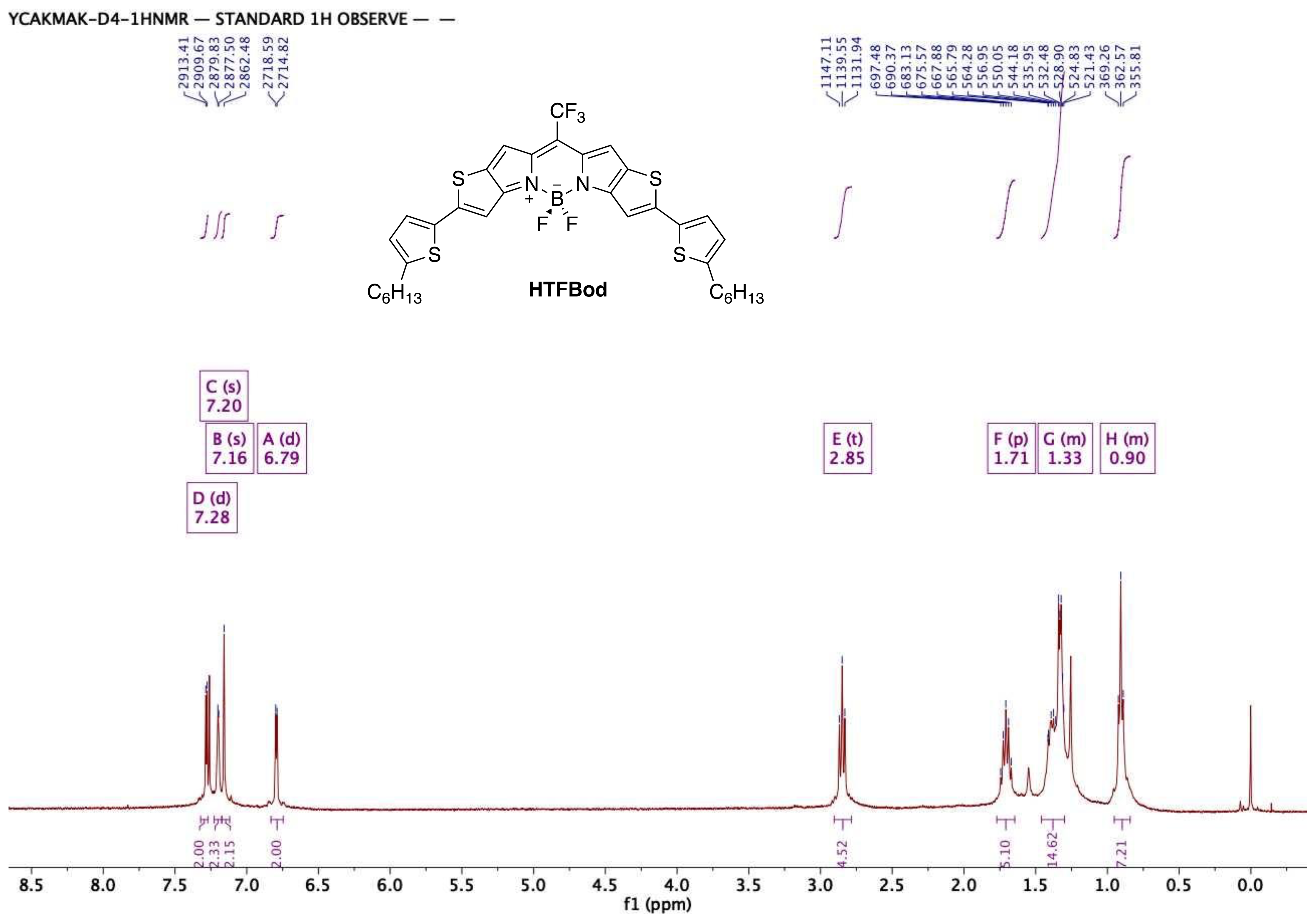

Supplement: Supplementary file 5 [file turkjchem-46-4-1120s5.tif]

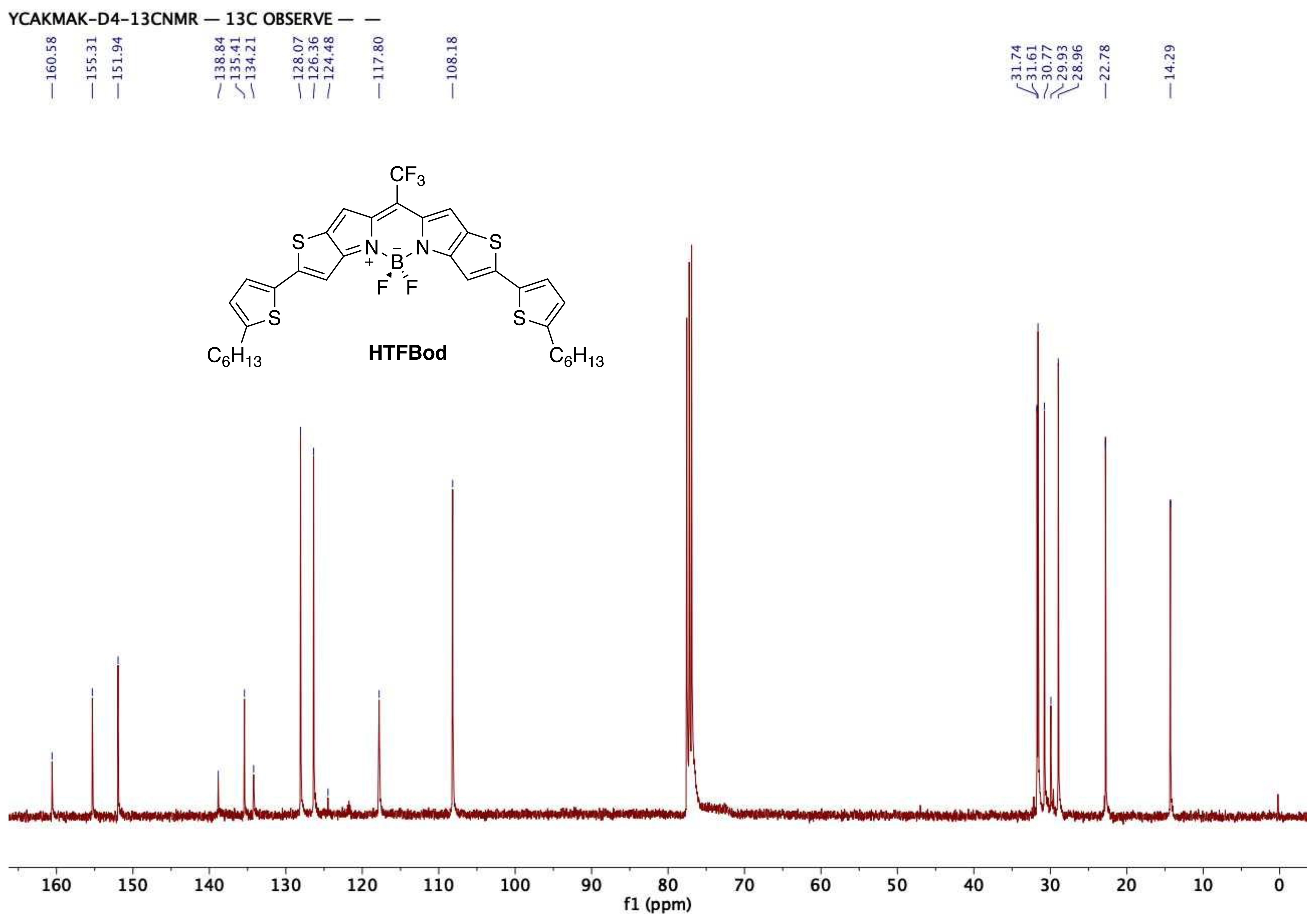

Supplement: Supplementary file 6 [file turkjchem-46-4-1120s6.tif]

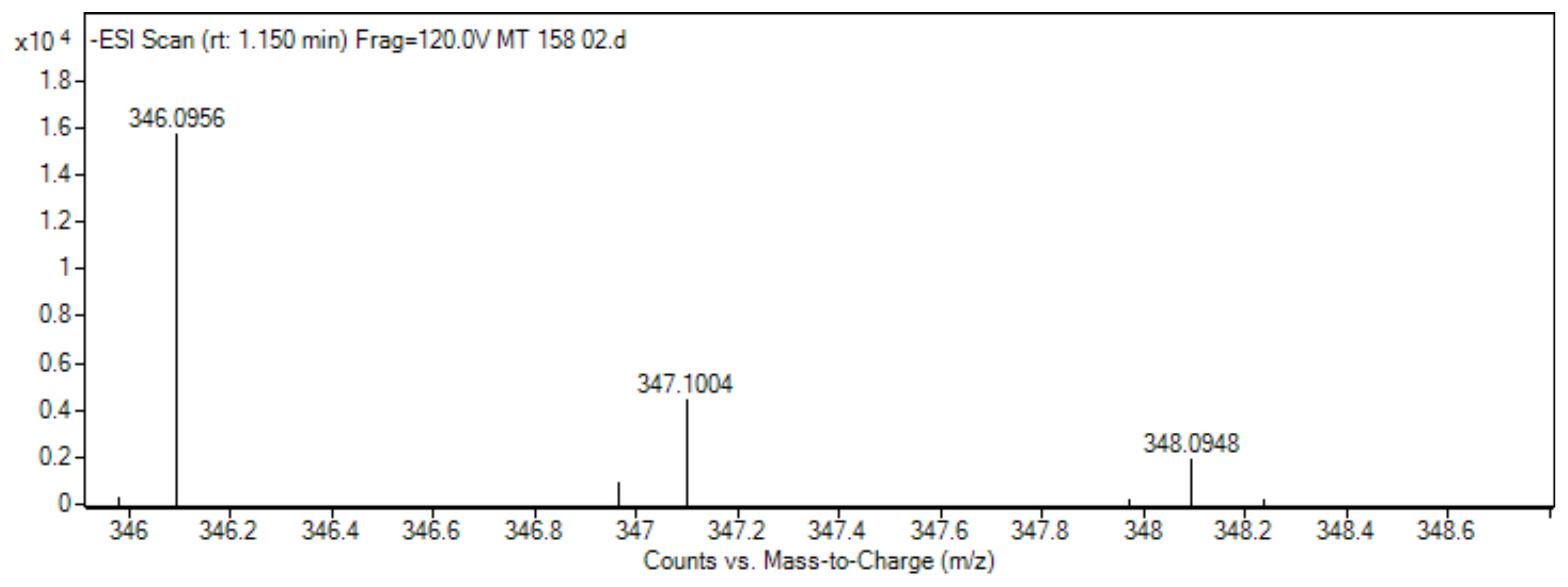

Supplement: Supplementary file 7 — ESI-HRMS spectrum of compound 4 [file turkjchem-46-4-1120s7.tif]

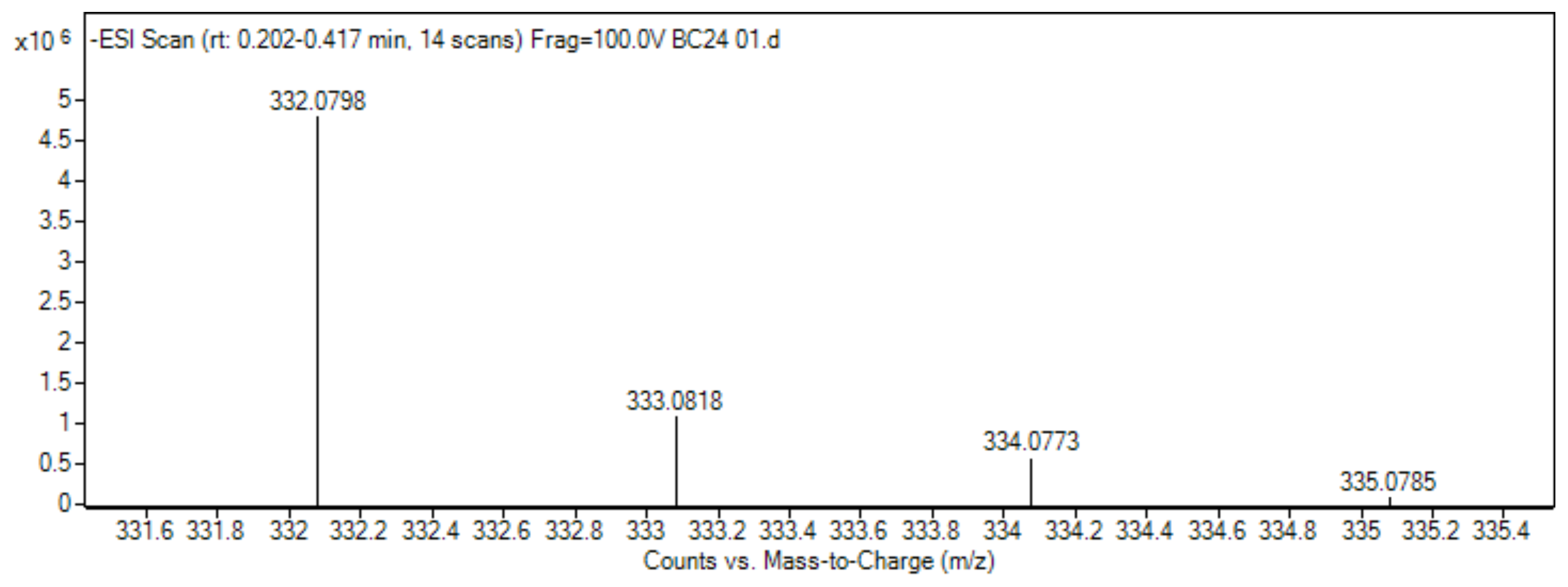

Supplement: Supplementary file 8 — ESI HRMS spectrum of compound 5 [file turkjchem-46-4-1120s8.tif]

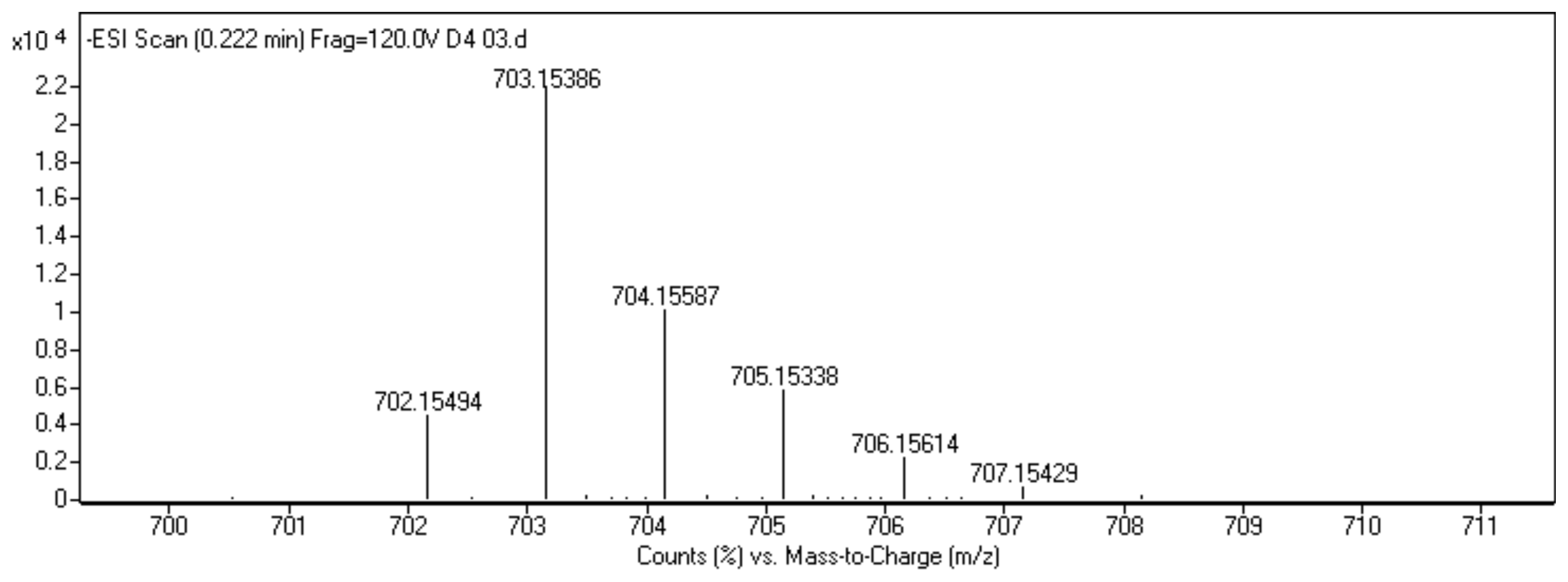

Supplement: Supplementary file 9 — ESI HRMS spectrum of compound HTFBod [file turkjchem-46-4-1120s9.tif]
